# Notch Signaling in Ischemic Damage and Fibrosis: Evidence and Clues from the Heart

**DOI:** 10.3389/fphar.2017.00187

**Published:** 2017-04-05

**Authors:** Silvia Nistri, Chiara Sassoli, Daniele Bani

**Affiliations:** ^1^Research Unit of Histology and Embryology, Department of Experimental and Clinical Medicine, University of FlorenceFlorence, Italy; ^2^Department of Experimental and Clinical Medicine, Section of Anatomy and Histology, University of FlorenceFlorence, Italy

**Keywords:** Notch, heart, fibrosis, ischemia-reperfusion, cardiac preconditioning

## Abstract

Notch signaling is a major intercellular coordination mechanism highly conserved throughout evolution. In vertebrates, Notch signaling is physiologically involved in embryo development, including mesenchymal cell commitment, formation of heart tissues and angiogenesis. In post-natal life, Notch signaling is maintained as a key mechanism of cell–cell communication and its dysregulations have been found in pathological conditions such as ischemic and fibrotic diseases. In the heart, Notch takes part in the protective response to ischemia, being involved in pre- and post-conditioning, reduction of reperfusion-induced oxidative stress and myocardial damage, and cardiomyogenesis. Conceivably, the cardioprotective effects of Notch may depend on neo-angiogenesis, thus blunting lethal myocardial ischemia, as well as on direct stimulation of cardiac cells to increase their resistance to injury. Another post-developmental adaptation of Notch signaling is fibrosis: being involved in the orientation of mesenchymal cell fate, Notch can modulate the differentiation of pro-fibrotic myofibroblasts, e.g., by reducing the effects of the profibrotic cytokine TGF-β. In conclusion, Notch can regulate the interactions between heart muscle and stromal cells and switch cardiac repair from a pro-fibrotic default pathway to a pro-cardiogenic one. These features make Notch signaling a suitable target for new cardiotropic therapies.

## Introduction

The Notch pathway is a major intercellular short-range coordination mechanism highly conserved throughout evolution and similar in all multicellular organisms from invertebrates to mammals (Kopan and Ilagan, 2009). Notch designates a trans-membrane receptor encoded by a gene originally identified as that responsible for the appearance of a ‘notch’ in the wings of *Drosophila melanogaster*. In mammals, four Notch receptors, 1–4, and five canonical ligands, Jagged 1–2 and Delta-like (DLL) 1,3 and 4, have been identified as membrane-spanning proteins (Guruharsha et al., 2012).

The mechanism of Notch signaling does not exploits the classical signal transduction pathways of most surface receptors: upon ligand binding, the surface metalloprotease ADAM10 clips the Notch extracellular domain just outside the plasma membrane and releases an extracellular Notch fragment which remains bound to its ligand and is then endocytosed by the ligand-bearing cell, which in turn undergoes signaling. Then, the inner membrane protease γ–secretase cleaves the Notch intracellular domain (NICD), the active form of Notch, which is released in the cytoplasm, migrates to the nucleus and binds to CSL transcription factors (also known as RBP-Jκ) regulating Notch target gene expression (Guruharsha et al., 2012). The encoded proteins regulate further expression of many downstream genes, some of which can either maintain the cell in an uncommitted state or induce differentiation, while others regulate cell proliferation and apoptosis (Miele et al., 2006). The increasing interest in Notch pathway ad a key regulator of cell function and differentiation has been paralleled by the development of appropriate methods and tools for its investigation in cellular and animal models, as exhaustively reported in a recent review (Zacharioudaki and Bray, 2014).

In Vertebrates, Notch signaling is physiologically involved in embryo development and morphogenesis which exploit its ability to mediate intercellular communication. In fact, the formation of distinct organs and tissues requires adhesion mechanisms which promote and maintain the sorting of different cell populations. In post-natal life, Notch signaling is maintained as a key mechanism of cell–cell communication and its dysregulations are implicated in tumor development and metastasis and in non-neoplastic pathological conditions such as ischemic and fibrotic diseases, sometimes playing a dual role as pathogenic mechanism or adaptive/compensatory response (Harper et al., 2003; Hori et al., 2013). In this context the heart, whose complex assembly requires the precise coordination of diverse cells, represents an appropriate paradigm to understand the roles of the Notch pathway in health and disease.

## Notch Pathway in the Developing and Diseased Heart

Notch signaling is a key mechanism of normal heart morphogenesis, being required for the formation of the atrioventricular canal and valves, outflow tract and coronary vessels, and for growth and differentiation of the endocardium, myocardium and epicardium (High and Epstein, 2008; Luxán et al., 2016). Among the cellular mechanisms operating during cardiac morphogenesis, Notch signaling has been shown to mediate epithelial-mesenchymal transition (EMT) of endocardial precursor cells: in particular, Notch signaling down-regulates surface cadherin expression and disables intercellular adhesion among these cells, allowing them to move, reach the atrio-ventricular and outflow tract regions, and pattern the cardiac valves (Timmerman et al., 2004). Moreover, during growth and three-dimensional organization of the ventricular myocardium, Notch signaling is required to sustain cardiomyocyte precursor proliferation and differentiation as well as compaction of the primitive trabecular myocardium (High and Epstein, 2008; Luxán et al., 2016). Finally, the Notch pathway is crucial for coronary vasculogenesis, namely the formation of primary vascular rudiments from the epicardial mesenchyme, and angiogenesis, namely the sprouting of new vessels from pre-existing ones. Both phenomena are regulated by vascular endothelial growth factor (VEGF), whose downstream pathway involves Notch/Jagged up-regulation by endothelial cells (Ferrara et al., 2003). Notch signaling appears a homeostatic regulator of the endothelium, since it can mediate either proliferation and resistance to apoptosis during active angiogenesis or contact inhibition and cell cycle arrest during blood vessel stabilization. In this latter phase, Notch also favors the recruitment of pericytes from the mesenchyme and stimulates growth, migration and resistance to apoptosis of vascular smooth muscle cells, thereby promoting the build-up of functional blood vessels (Sainson and Harris, 2008).

The pivotal role of the Notch pathway in heart morphogenesis emerges from both studies on Notch or Jagged1 knock-out mice and clinical reports, showing that defective Notch signaling is correlated with cardiac malformations such as Tetralogy of Fallot in Alagille syndrome, aberrant bicuspid aortic valve and left ventricular non-compaction cardiomyopathy (High and Epstein, 2008; Luxán et al., 2016). In the adult heart, Notch signaling between mature cells is absent under physiological conditions but can be roused to take part in the protective response to injury, as later discussed.

Another morphogenetic effect of the Notch pathway with major repercussions on the adult diseased heart is the regulation of stromal cell differentiation and extracellular matrix (ECM) production (Hu and Phan, 2016). In the embryo, Notch signaling promotes EMT and generates mesenchymal cells: in turn, these cells differentiate into different stromal cell lineages, including the cardiac valves and coronary endothelium. Although in adult tissues EMT is suspected to play some role in the generation of new fibroblasts and myofibroblasts, the main pro-fibrotic cells, during the development of organ fibrosis (Hu and Phan, 2016), the existing evidence suggests that Notch signaling can rather exert anti-fibrotic effects on the diseased heart, for instance by counteracting the pro-fibrotic cytokine transforming growth factor (TGF)-β and reducing myofibroblast proliferation (Nemir et al., 2014).

## Notch Pathway in the Ischemic Heart

Myocardial infarction (MI), one of the leading causes of death worldwide, mainly depends on coronary artery occlusion and ischemia followed by reperfusion (I/R), in which blood flow restoration is accompanied by oxidative stress exacerbating myocardial damage. Noteworthy, the adult myocardium can re-express fetal genes as an adaptive response to injury: in this context, increased Notch1 signaling was demonstrated in surviving cardiomyocytes of the MI border zone (Gude et al., 2008). Several studies have shown that Notch signaling protects the heart from I/R-induced myocardial injury: activation of Notch1 pathway limits the extent of ischemic damage, promotes coronary neo-angiogenesis and revascularization of the ischemic myocardium, reduces myocardial fibrosis and improves heart function (Gude et al., 2008; Kratsios et al., 2010; Li et al., 2011; Ferrari and Rizzo, 2014). Conversely, in systemic Notch1 deficient mice, I/R leads to the development of a larger myocardial infarct area and worsening of heart function than wild-type controls (Li et al., 2011).

Notch1 plays an important role in the protection of ischemic myocardium during pre- (IPC) and post-conditioning (IPost), well-known adaptive responses of the heart to increase its resistance to I/R injury (Zhao et al., 2003). Notch1 pathway is activated during myocardial IPC and IPost and leads to reduction of cardiomyocyte apoptosis, infarct size and contractile impairment. Conversely, inhibition of Notch1 signaling by N1ICD knockdown abrogates IPC- and IPost-induced cardioprotection (Zhou et al., 2013).

**FIGURE 1:**
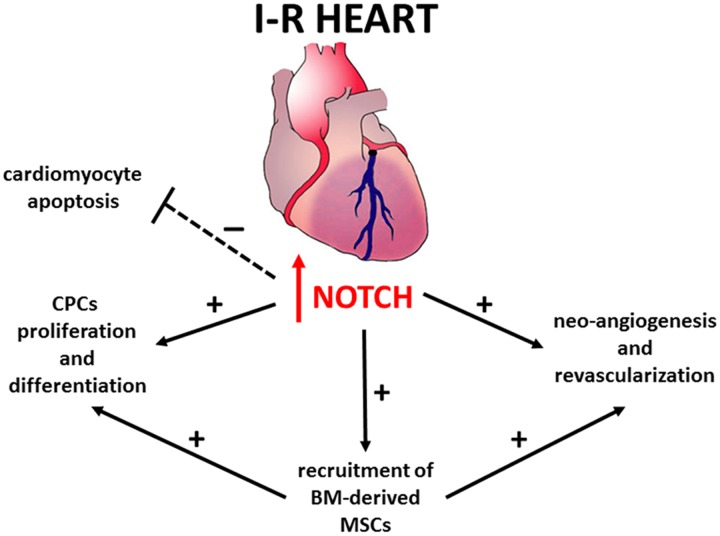
**Notch signaling in repair and regeneration of the ischemic and reperfused heart**.

The mechanisms underlying Notch-mediated cardio-protection are complex and involve an interplay between mature and immature cardiomyocytes, cardiac progenitors cells (CPCs) and bone marrow (BM)-derived cells (**Figure 1**). Notch1 prevents cardiomyocyte apoptosis by activation of PI3K/AKT pro-survival signaling and regulation of apoptotic genes (Kratsios et al., 2010; Pei et al., 2013; Zhou et al., 2013). Moreover, Notch signaling induces cell cycle re-entry of immature cardiomyocytes (Campa et al., 2008; Sassoli et al., 2011), promotes proliferation and myogenic differentiation of CPCs (Boni et al., 2008), decreases oxidative/nitrosative stress (Pei et al., 2013) and prevents cardiac fibrosis (Fan et al., 2011).

Recent studies indicate that Notch1 is ineffective in promoting cardiac regeneration in adults due to permanent epigenetic modifications at the Notch-responsive promoters which render their transcriptional repression irreversible (Felician et al., 2014). Consistently, in cardiac-specific Notch1 deficient mice, the loss of Notch1 in post-natal cardiomyocytes did not affect the severity of myocardial injury (Li et al., 2011). Hence, the beneficial effects of Notch1 re-activation previously observed in transgenic animals (Kratsios et al., 2010) could be mediated *via* other cell types, such as CPCs and BM-derived cells. Accordingly, Notch1 recruits BM-derived mesenchymal stem cells (MSCs) in the infarction border zone, promoting proliferation and preventing apoptosis (Li et al., 2011). Moreover, transplantation of N1ICD-overexpressing MSCs reduces – while that of Notch1-deficient MSCs increases – both infarct size and contractile impairment (Li et al., 2011). Overall, these findings suggest that the maintenance or reactivation of Notch signaling in cardiac cells can be a therapeutic target to protect against myocardial damage.

## Notch Pathway in Cardiac Fibrosis

Cardiac fibrosis, a late complication of many heart diseases, can occur as myocardial replacement fibrosis to prevent cardiac rupture, for instance after MI, or as interstitial fibrosis without cardiomyocyte loss, an adaptation to chronic injury by functional overload, ischemia and cardiomyopathies. Started as compensatory to organ damage, cardiac fibrosis becomes maladaptive and dysfunctional in the long term (Rockey et al., 2015; Travers et al., 2016). In general, fibrosis results from an imbalance between ECM synthesis and degradation by fibrogenic cells, chiefly myofibroblasts. In response to pro-inflammatory and pro-fibrotic mediators up-regulated in cardiac injury, among which TGF-β1 plays a major role, resident cardiac fibroblasts, CD45+ hemopoietic stromal cells and, perhaps, EMT-derived fibroblasts, vascular pericytes and immune cells are recruited and prompted to differentiate into myofibroblasts, characterized by dual immunophenotypical and ultrastructural features of fibroblasts and smooth muscle cells (Bani and Nistri, 2014; Ivey and Tallquist, 2016; Pinto et al., 2016). Myofibroblast contraction and excess ECM deposition cause the distortion of the normal myocardial architecture. Moreover, myofibroblasts secrete a variety of mediators which stimulate autocrine cell activation and fibrogenesis and exert paracrine effects on the cells nearby, causing chronic inflammation and further cardiomyocyte dysfunction (Travers et al., 2016).

Several studies have shown that Notch signaling is involved in counteracting cardiac fibrosis, primarily via inhibition of myofibroblast differentiation. In particular, the expression of Notch1, 3, and 4 are down-regulated during fibroblast–myofibroblast transition in neonatal hearts, while Notch signaling inhibition promotes myofibroblast formation (Fan et al., 2011). Consistently, in a mouse model of pressure overload, Notch1 controlled the balance between fibrotic and regenerative repair by inhibiting myofibroblast proliferation and promoting mobilization and expansion of cardiac muscle precursor cells (Nemir et al., 2014). Recently, *in vivo* intramyocardial delivery of hydrogels containing the Notch1 ligand Jagged-1 in rats with MI reduced cardiac fibrosis (Boopathy et al., 2015). Moreover, augmentation of Notch3 expression by lentiviral transfection inhibited fibroblast–myofibroblast transition both in TGF-β1-treated cardiac fibroblasts *in vitro* and in mice with MI, minimizing cardiac fibrosis (Zhang et al., 2016). As previously mentioned, Notch signaling can inhibit EMT (Zhou et al., 2015; Hu and Phan, 2016), which also contributes to cardiac fibrosis (von Gise and Pu, 2012). This point, however, remains controversial: indeed, in Notch transgenic mice undergoing MI and pressure overload, Notch induced epicardial cells to undergo EMT and generate a multipotent stromal cell population capable of differentiating into fibroblasts and producing reparative fibrosis (Russell et al., 2011).

**FIGURE 2:**
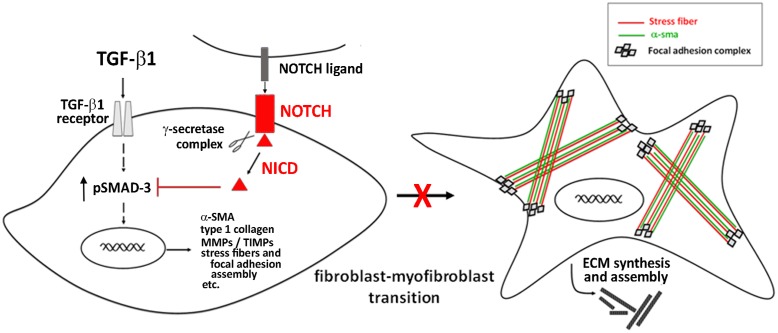
**Notch signaling in the regulation of cardiac fibrosis**.

The main identified mechanism by which Notch signaling interferes with myofibroblast differentiation consists in its ability to antagonize TGF-β/Smad3 signaling, the key intracellular pathway promoting cell activation and fibrogenesis (Zhang et al., 2016; Travers et al., 2016) (**Figure 2**).

Targeting the Notch pathway may be a meaningful therapeutic strategy for cardiac fibrosis. In this context, the hormone relaxin, known as anti-fibrotic agent and under clinical investigation in heart failure patients (Moin et al., 2016), was shown to inhibit the TGF-β1/Smad3 axis and to counteract TGF-β1-induced transition of neonatal cardiac stromal cells and NIH3T3 fibroblasts to myofibroblasts acting *via* the up-regulation of Notch1 signaling (Sassoli et al., 2013; Squecco et al., 2015; Zhou et al., 2015).

## Conclusion

The Notch pathway is pivotal in the protection and healing of the ischemic heart because it regulates key mechanisms of myocardial resistance to ischemia and controls the balance between fibrotic and regenerative repair. Targeting Notch signaling, for example by soluble Notch ligands or Notch pathway activating molecules delivered intramyocardially or embedded into suitable biomaterials (Gude et al., 2008; Limana et al., 2013; Boccalini et al., 2015; Boopathy et al., 2015), may be a worthwhile therapeutic approach to cardiovascular disease.

## Author Contributions

SN conceived the manuscript and wrote the chapter on cardiac ischemia-reperfusion, CS wrote the chapter on cardiac fibrosis, DB wrote the Introduction and the chapter on heart embryology. All authors contributed equally to conceiving and drawing of figures.

## Conflict of Interest Statement

The authors declare that the research was conducted in the absence of any commercial or financial relationships that could be construed as a potential conflict of interest.

## References

[B1] Bani D. Nistri S. (2014). New insights into the morphogenic role of stromal cells and their relevance for regenerative medicine. lessons from the heart. *J. Cell. Mol. Med.* 18 363–370. 10.1111/jcmm.12247 24533677 PMC3955144

[B2] Boccalini G. Sassoli C. Formigli L. Bani D. Nistri S. (2015). Relaxin protects cardiac muscle cells from hypoxia/reoxygenation injury: involvement of the Notch-1 pathway. *FASEB J.* 29 239–249. 10.1096/fj.14-254854 25342127

[B3] Boni A. Urbanek K. Nascimbene A. Hosoda T. Zheng H. Delucchi F. (2008). Notch1 regulates the fate of cardiac progenitor cells. *Proc. Natl. Acad. Sci. U.S.A.* 105 15529–15534. 10.1073/pnas.0808357105 18832173 PMC2563067

[B4] Boopathy A. V. Martinez M. D. Smith A. W. Brown M. E. García A. J. Davis M. E. (2015). Intramyocardial delivery of notch ligand-containing hydrogels improves cardiac function and angiogenesis following infarction. *Tissue Eng. Part A* 21 2315–2322. 10.1089/ten.TEA.2014.0622 25982380 PMC4555480

[B5] Campa V. M. Gutiérrez-Lanza R. Cerignoli F. Díaz-Trelles R. Nelson B. Tsuji T. (2008). Notch activates cell cycle reentry and progression in quiescent cardiomyocytes. *J. Cell Biol.* 183 129–141. 10.1083/jcb.200806104 18838555 PMC2557048

[B6] Fan Y. H. Dong H. Pan Q. Cao Y. J. Li H. Wang H. C. (2011). Notch signaling may negatively regulate neonatal rat cardiac fibroblast-myofibroblast transformation. *Physiol. Res.* 60 739–748. 21812518 10.33549/physiolres.932149

[B7] Felician G. Collesi C. Lusic M. Martinelli V. Ferro M. D. Zentilin L. (2014). Epigenetic modification at Notch responsive promoters blunts efficacy of inducing notch pathway reactivation after myocardial infarction. *Circ. Res.* 115 636–649. 10.1161/CIRCRESAHA.115.304517 25114098

[B8] Ferrara N. Gerber H. P. LeCouter J. (2003). The biology of VEGF and its receptors. *Nat. Med.* 9 669–676. 10.1038/nm0603-669 12778165

[B9] Ferrari R. Rizzo P. (2014). The Notch pathway: a novel target for myocardial remodeling therapy? *Eur. Heart J.* 35 2140–2145. 10.1093/eurheartj/ehu244 24970336

[B10] Gude N. A. Emmanuel G. Wu W. Cottage C. T. Fischer K. Quijada P. (2008). Activation of Notch-mediated protective signaling in the myocardium. *Circ. Res.* 102 1025–1035. 10.1161/CIRCRESAHA.107.164749 18369158 PMC3760732

[B11] Guruharsha K. G. Kankel M. W. Artavanis-Tsakonas S. (2012). The Notch signalling system: recent insights into the complexity of a conserved pathway. *Nat. Rev. Genet.* 13 654–666. 10.1038/nrg3272 22868267 PMC4369923

[B12] Harper J. A. Yuan J. S. Tan J. B. Visan I. Guidos C. J. (2003). Notch signaling in development and disease. *Clin. Genet.* 64 461–472. 10.1046/j.1399-0004.2003.00194.x 14986825

[B13] High F. A. Epstein J. A. (2008). The multifaceted role of Notch in cardiac development and disease. *Nat. Rev. Genet.* 9 49–61. 10.1038/nrg2279 18071321

[B14] Hori K. Sen A. Artavanis-Tsakonas S. (2013). Notch signaling at a glance. *J. Cell Sci.* 15 2135–2140. 10.1242/jcs.127308 PMC3672934 23729744

[B15] Hu B. Phan S. H. (2016). Notch in fibrosis and as a target of anti-fibrotic therapy. *Pharmacol. Res.* 108 57–64. 10.1016/j.phrs.2016.04.010 27107790 PMC5074914

[B16] Ivey M. J. Tallquist M. D. (2016). Defining the cardiac fibroblast. *Circ. J.* 80 2269–2276. 10.1253/circj.CJ-16-1003 27746422 PMC5588900

[B17] Kopan R. Ilagan M. X. (2009). The canonical Notch signaling pathway: unfolding the activation mechanism. *Cell* 137 216–233. 10.1016/j.cell.2009.03.045 19379690 PMC2827930

[B18] Kratsios P. Catela C. Salimova E. Huth M. Berno V. Rosenthal N. (2010). Distinct roles for cell-autonomous Notch signaling in cardiomyocytes of the embryonic and adult heart. *Circ. Res.* 106 559–572. 10.1161/CIRCRESAHA.109.203034 20007915

[B19] Li Y. Hiroi Y. Ngoy S. Okamoto R. Noma K. Wang C. Y. (2011). Notch1 in bone marrow-derived cells mediates cardiac repair after myocardial infarction. *Circulation* 123 866–876. 10.1161/CIRCULATIONAHA.110.947531 21321153 PMC3488350

[B20] Limana F. Esposito G. Fasanaro P. Foglio E. Arcelli D. Voellenkle C. (2013). Transcriptional profiling of HMGB1-induced myocardial repair identifies a key role for Notch signaling. *Mol. Ther.* 21 1841–1851. 10.1038/mt.2013.137 PMC3808142 23760446

[B21] Luxán G. D’Amato G. MacGrogan D. de la Pompa J. L. (2016). Endocardial Notch signaling in cardiac development and disease. *Circ. Res.* 118 e1–e18. 10.1161/CIRCRESAHA.115.305350 26635389

[B22] Miele L. Golde T. Osborne B. (2006). Notch signaling in cancer. *Curr. Mol. Med.* 6 905–918. 10.2174/156652406779010830 17168741

[B23] Moin D. S. Bloom M. W. Papadimitriou L. Butler J. (2016). Serelaxin for the treatment of heart failure. *Expert Rev. Cardiovasc. Ther.* 14 667–675. 10.1586/14779072.2016.1174067 27045761

[B24] Nemir M. Metrich M. Plaisance I. Lepore M. Cruchet S. Berthonneche C. (2014). The Notch pathway controls fibrotic and regenerative repair in the adult heart. *Eur. Heart J.* 35 2174–2185. 10.1093/eurheartj/ehs269 23166366 PMC4139705

[B25] Pei H. Yu Q. Xue Q. Guo Y. Sun L. Hong Z. (2013). Notch1 cardioprotection in myocardial ischemia/reperfusion involves reduction of oxidative/nitrative stress. *Basic Res. Cardiol.* 108:373 10.1007/s00395-013-0373-x 23989801

[B26] Pinto A. R. Ilinykh A. Ivey M. J. Kuwabara J. T. D’Antoni M. L. Debuque R. (2016). Revisiting cardiac cellular composition. *Circ. Res.* 118 400–409. 10.1161/CIRCRESAHA.115.307778 26635390 PMC4744092

[B27] Rockey D. C. Bell P. D. Hill J. A. (2015). Fibrosis - A common pathway to organ injury and failure. *N. Engl. J. Med.* 372 1138–1149. 10.1056/NEJMra1300575 25785971

[B28] Russell J. L. Goetsch S. C. Gaiano N. R. Hill J. A. Olson E. N. Schneider J. W. (2011). A dynamic notch injury response activates epicardium and contributes to fibrosis repair. *Circ. Res.* 108 51–59. 10.1161/CIRCRESAHA.110.233262 21106942 PMC3042596

[B29] Sainson R. A. Harris A. L. (2008). Regulation of angiogenesis by homotypic and heterotypic notch signaling in endothelial cells and pericytes: from basic research to potential therapies. *Angiogenesis* 11 41–51. 10.1007/s10456-008-9098-0 18256896

[B30] Sassoli C. Chellini F. Pini A. Tani A. Nistri S. Nosi D. (2013). Relaxin prevents cardiac fibroblast-myofibroblast transition via notch-1-mediated inhibition of TGF-β/Smad3 signaling. *PLoS ONE* 8:e63896 10.1371/journal.pone.0063896 PMC3660557 23704950

[B31] Sassoli C. Pini A. Mazzanti B. Quercioli F. Nistri S. Saccardi R. (2011). Mesenchymal stromal cells affect cardiomyocyte growth through juxtacrine Notch-1/Jagged-1 signaling and paracrine mechanisms: clues for cardiac regeneration. *J. Mol. Cell. Cardiol.* 51 399–408. 10.1016/j.yjmcc.2011.06.004 21712044

[B32] Squecco R. Sassoli C. Garella R. Chellini F. Idrizaj E. Nistri S. (2015). Inhibitory effects of relaxin on cardiac fibroblast-to-myofibroblast transition: an electrophysiological study. *Exp. Physiol.* 100 652–666. 10.1113/EP085178 25786395

[B33] Timmerman L. A. Grego-Bessa J. Raya A. Bertrán E. Pérez-Pomares J. M. Díez J. (2004). Notch promotes epithelial-mesenchymal transition during cardiac development and oncogenic transformation. *Genes Dev.* 18 99–115. 10.1101/gad.276304 14701881 PMC314285

[B34] Travers J. G. Kamal F. A. Robbins J. Yutzey K. E. Blaxall B. C. (2016). Cardiac fibrosis: the fibroblast awakens. *Circ. Res.* 118 1021–1040. 10.1161/CIRCRESAHA.115.306565 26987915 PMC4800485

[B35] von Gise A. Pu W. T. (2012). Endocardial and epicardial epithelial to mesenchymal transitions in heart development and disease. *Circ. Res.* 110 1628–1645. 10.1161/CIRCRESAHA.111.259960 22679138 PMC3427736

[B36] Zacharioudaki E. Bray S. J. (2014). Tools and methods for studying Notch signaling in *Drosophila melanogaster*. *Methods* 68 173–182. 10.1016/j.ymeth.2014.03.029 24704358 PMC4059942

[B37] Zhang M. Pan X. Zou Q. Xia Y. Chen J. Hao Q. (2016). Notch3 ameliorates cardiac fibrosis after myocardial infarction by inhibiting the TGF-β1/Smad3 pathway. *Cardiovasc. Toxicol.* 16 316–324. 10.1007/s12012-015-9341-z 26487518

[B38] Zhao Z. Q. Corvera J. S. Halkos M. E. Kerendi F. Wang N. P. Guyton R. A. (2003). Inhibition of myocardial injury by ischemic postconditioning during reperfusion: comparison with ischemic preconditioning. *Am. J. Physiol. Heart Circ. Physiol.* 285 H579–H588. 10.1152/ajpheart.01064.2002 12860564

[B39] Zhou X. Chen X. Cai J. J. Chen L. Z. Gong Y. S. Wang L. X. (2015). Relaxin inhibits cardiac fibrosis and endothelial-mesenchymal transition via the Notch pathway. *Drug Des. Devel. Ther.* 9 4599–4611. 10.2147/DDDT.S85399 PMC4541540 26316699

[B40] Zhou X. L. Wan L. Xu Q. R. Zhao Y. Liu J. C. (2013). Notch signaling activation contributes to cardioprotection provided by ischemic preconditioning and postconditioning. *J. Transl. Med.* 11:251 10.1186/1479-5876-11-251 PMC3853230 24098939

